# Mindfulness-based interventions in epilepsy: a systematic review

**DOI:** 10.1186/s12883-017-0832-3

**Published:** 2017-03-20

**Authors:** Karen Wood, Maggie Lawrence, Bhautesh Jani, Robert Simpson, Stewart W. Mercer

**Affiliations:** 1 General Practice and Primary Care, Institute of Health and Wellbeing, University of Glasgow, 1 Horselethill Road, Glasgow, G12 9LX Scotland UK; 2 Institute for Applied Health Research, School of Health and Life Sciences Glasgow Caledonian University, Glasgow, G4 0BA Scotland, UK

**Keywords:** Mindfulness, Epilepsy, Systematic review, Stress, Anxiety, Depression

## Abstract

**Background:**

Mindfulness based interventions (MBIs) are increasingly used to help patients cope with physical and mental long-term conditions (LTCs). Epilepsy is associated with a range of mental and physical comorbidities that have a detrimental effect on quality of life (QOL), but it is not clear whether MBIs can help. We systematically reviewed the literature to determine the effectiveness of MBIs in people with epilepsy.

**Methods:**

Medline, Cochrane Central Register of Controlled Trials, EMBASE, CINAHL, Allied and Complimentary Medicine Database, and PsychInfo were searched in March 2016. These databases were searched using a combination of subject headings where available and keywords in the title and abstracts. We also searched the reference lists of related reviews. Study quality was assessed using the Cochrane Collaboration risk of bias tool.

**Results:**

Three randomised controlled trials (RCTs) with a total of 231 participants were included. The interventions were tested in the USA (*n* = 171) and China (Hong Kong) (*n* = 60). Significant improvements were reported in depression symptoms, quality of life, anxiety, and depression knowledge and skills. Two of the included studies were assessed as being at unclear/high risk of bias - with randomisation and allocation procedures, as well as adverse events and reasons for drop-outs poorly reported. There was no reporting on intervention costs/benefits or how they affected health service utilisation.

**Conclusion:**

This systematic review found limited evidence for the effectiveness of MBIs in epilepsy, however preliminary evidence suggests it may lead to some improvement in anxiety, depression and quality of life. Further trials with larger sample sizes, active control groups and longer follow-ups are needed before the evidence for MBIs in epilepsy can be conclusively determined.

**Electronic supplementary material:**

The online version of this article (doi:10.1186/s12883-017-0832-3) contains supplementary material, which is available to authorized users.

## Background

The prevalence of stress, anxiety, and depression is higher among people with epilepsy when compared with the general population, and suicide rates are similarly elevated [1–5]. The prevalence of anxiety among people with epilepsy is estimated to be between 10 and 25% [5], whilst Fiest et al. [6] reported a 13% lifetime prevalence of depression.

Stress is widely recognised as a risk factor for developing both anxiety and depression, and may also be a trigger for seizures in people with epilepsy [7]. For example, epileptics report stress as one of the main precipitants of seizures [7, 8], and chronic stress has been linked to a higher frequency of seizures [9–12]. The unpredictability of recurrent seizures and a resultant feeling of lack of control can be stressful, as can concern about potential for injury during a seizure [7, 13–15]. Moreover, anti-epileptic medications can have unpleasant side effects [14, 16, 17], and having a diagnosis of epilepsy can limit an individual’s employment opportunities and render them unable to drive [7, 14]. Comorbid anxiety, depression, and stress negatively influence quality of life (QOL) in people with epilepsy [3, 4] and are likely to be under-reported, under-diagnosed, and under-treated [18, 19].

Psychological therapies can form a useful component of treatment of mental health disorders in epilepsy [20, 21]. For example, a recent systematic review [22] found that “*…psychotherapy can improve depression and anxiety in patients with epilepsy*”. In this context, Tang et al. [21] describe the primary aim of psycho-behavioural therapies in epilepsy to improve the ability of the individual to cope with their illness, but additionally note they may improve “*seizure control and psychological well-being*”. A commonly used psychological treatment is Cognitive Behavioural Therapy (CBT). A systematic review of CBT for people with epilepsy suggested that CBT interventions which are orientated towards treating depression rather than seizure control are “*likely efficacious*” and supported the use of CBT in some “*clinical practice recommendations*” [23].

Mindfulness-based interventions (MBIs) are increasingly used to treat stress and mental health comorbidities in people with long-term conditions (LTCs), including other neurological conditions such as multiple sclerosis [24] and stroke [25]. Mindfulness interventions are typically based on Mindfulness Based Stress Reduction (MBSR) which has three core meditation practices (the ‘body scan’, ‘following the breath’ and ‘mindful movement’) [26, 27]. The highest quality evidence for MBIs is for recurrent depression [28–30]. MBIs could potentially offer a further treatment option for people with epilepsy; however the effectiveness of MBIs for this group has not previously been reviewed. An important safety consideration is that case reports exist suggesting that meditation training might be associated with an increased risk of seizures in known epileptics. This is thought due to neuronal hypersynchrony arising from meditation practise, however, currently this phenomenon remains poorly characterised and remains theoretical [31]. This paper aimed to evaluate the effectiveness of MBIs in people with epilepsy by means of a systematic review of the evidence.

## Methods

### Protocol

A protocol for this review is available [32].

### Selection criteria

To enable the identification and selection of the best available evidence regarding the therapeutic benefits of MBIs for people with epilepsy we determined inclusion criteria relating to Population, Interventions, Comparator and Outcomes (PICO) (Table 1).

**Table 1:** PICO criteria

Population	Adult patients (aged 18 years or older) with a clinical diagnosis of epilepsy will be included.
Interventions	Trials which solely focus on, or incorporate a Mindfulness intervention where the mindfulness data can be extracted, will be selected. As with many other mind-body interventions, Mindfulness as a therapeutic intervention is inherently varied and heterogeneous. Thus different forms, duration and frequency of Mindfulness interventions will be included [45].
Comparator	Usual care or any active comparator
Outcome measures	Confidence
	Well-being
	Anxiety depression
	Social participation
	Perceived self-health
	Quality of Life
	Physiological outcomes e.g., blood pressure
	Seizure frequency; seizure duration

### Search strategy

In March 2016 a systematic search for published and unpublished studies was conducted in six major electronic bibliographic databases: Cochrane Central Register of Controlled Trials, MEDLINE, EMBASE, CINAHL, Allied and Complementary Medicine Database, and PsycInfo. Selected medical subject headings were combined with key words relating to epilepsy and mindfulness to create a search strategy, which was finalised for use in MEDLINE (Additional file 1) and amended for use in the other databases, using appropriate controlled vocabulary, Boolean operators, and search symbols. The databases searched retrieve conference abstracts, theses and dissertations; in addition key words were used to conduct a basic search of Google. Delimiters were: dates searched (1980–2016); research subjects (human); and language (English). RefWorks was used to store and manage the results of the database searches.

### Selection of papers for inclusion

The bibliographic records identified by the searches were screened for relevance using broad inclusion criteria, i.e., ‘epilepsy’ and ‘mindfulness’. All relevant papers were then screened, using the PICO inclusion criteria (Table 1), to select eligible papers. All selected papers were subject to methodological appraisal. Methodological quality was assessed using the Cochrane Risk of Bias tool [33]. Quality was assessed as being of low/unclear/high risk of bias against seven criteria: random sequence generation (selection bias), allocation concealment (selection bias), blinding of assessors (performance bias), blinding of outcome assessment (detection bias), incomplete outcome data (attrition bias), selective reporting (reporting bias), and ‘other’. Due to the paucity of available evidence, no papers were excluded on the grounds of quality. However, methodological issues are discussed below and reported in the evidence table.

### Data extraction

**Fig. 1:**
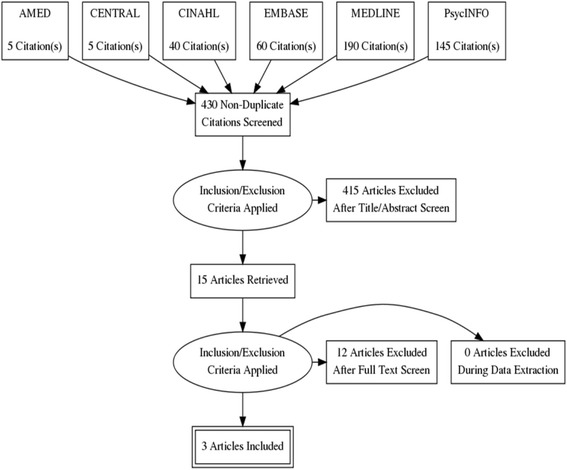
Flow Diagram

Data, including details of study design and methods, interventions (delivery and content), populations, and primary and secondary outcomes, were extracted from papers using a data extraction tool developed by the authors. All screening and assessment processes for each paper were conducted by two reviewers who worked independently and then met to discuss and agree the outcomes; any disagreements were resolved consensually (Fig. 1).

### Data synthesis

Due to the heterogonous nature of the papers included in the review, meta-analyses were not possible; therefore, the review findings are presented in narrative form.

### Results

The three studies included in the review were based in the USA - involving 53 participants [34] and 118 participants [35] - and also in China (Hong Kong) involving 60 participants [36]. Most participants were female and mean age varied from 34.77 years (intervention group [36]) to 41.2 years (all participants [35]). The majority of participants were White/Caucasian in the two studies which reported ethnicity [34, 35] (Table 2). All participants had been diagnosed with epilepsy; those in the Tang et al. [36] study were required to have a diagnosis of epilepsy resistant to antiepileptic drug treatment, and the study outlined participants’ type of epilepsy, mean duration of diagnosis, age at diagnosis, number and type of epileptic medications, seizure frequency, last seizure and co-morbidities. However, information pertaining to participants’ epilepsy was poorly reported in Thompson et al. [34] and not at all by Thompson et al. [35].

**Table 2:** Participant characteristics

	Thompson et al. (2010) [34]		Thompson et al. (2015) [35]		Tang et al. (2015) [36]	
	Intervention	Control	Intervention	Control	Intervention	Control
Number of Participants	26	27	62	56	30	30
Number of participants (% female)	20 (77%)	23 (85%)	-- (65.3%)		16 (53.3%)	16 (53.3%)
Mean age (SD)	36.4 years	35.4 years	41.2 years		34.77 years (10.26)	35.47 years (11.22)
Ethnicity	20 (77%) White 6 (23%) African American	19 (70%) White 7 (26%) African American 1 (4%) Other	70 (59.3%) Caucasian 14 (11.9%) African American 3 (2.5%) Hispanic 6 (5.1%) Other race		NR	NR
Socio-economic status	NR	NR	NR		NR	NR
Employment status	12 (52%) Not working/retired 7 (30%) Full Time 3 (13%) Part Time 1 (4%) Student	12 (50%) Not working/retired 6 (25%) Full Time 3 (13%) Part Time 3 (13%) Student	NR		56.7% Full Time	53.3% Full Time
Educational status	7 (30%) High School or less 6 (26%) Completed some college 9 (39%) Graduated college or more	6 (25%) High School or less 9 (38%) Completed some college 9 (38%) Graduated college or more	NR		76.7% (>11 years of education)	80% (>11 years of education)
Living arrangement	17 (74%) Immediate Family 3 (13%) Alone 2 (9%) Friend/roommate/partner	16 (67%) Immediate family 2 (8%) Alone 6 (25%) Friend/roommate/partner	NR		NR	NR
Medications	9 (39%) Antidepressants	9 (37%) Antidepressants	30.5% Antidepressants		50% Carbamazepine 30% Valproate 30% Levetiracetam 26.7% Lamotrigine	40% Carbamazepine 36.7% Valproate 26.7% Levetiracetam 26.7% Lamotrigine
Co-morbidities	10 (44%) MDD	9 (38%) MDD	NR		3 (10%) Concomitant Psychiatric Illness 5 (16.7%) Concomitant Non-Psychiatric Illness	2 (6.7%) Concomitant Psychiatric Illness 5 (16.7%) Concomitant Non-Psychiatric Illness
Mood and Quality of Life Baseline Measures						
mBDI	31.1	27.9	20.3	20.2	N/A	N/A
BDI	15.2	12.4	7.3	7.2	N/A	N/A
NDDI-E	N/A	N/A	13.1	13.0	N/A	N/A
PHQ-9	N/R	N/R	6.7	5.8	N/A	N/A
QOLIE-31-P	N/A	N/A	N/A	N/A	57.14	59.34
BAI	N/A	N/A	N/A	N/A	15.10	13.53
BDI-II	N/A	N/A	N/A	N/A	12.43	13.53

*BAI* Beck Anxiety Inventory, *BDI* Beck Depression Inventory, *BDI-II* Beck Depression Inventory II, *MDD* Major Depressive Disorder, *mBDI* modified Beck Depression Inventory, *NDDI-E* Neurological Disorders Depression Inventory for Epilepsy, *N/A* Not Applicable, *NR* Not Reported, *PHQ-9* Patient Health Questionnaire 9, *QOLIE-31-P* Quality of Life in Epilepsy Inventory

Participant baseline characteristics were reported separately for intervention and control groups in Thompson et al. [34] and Tang et al. [36] but for participants as a whole in Thompson et al. [35] (and not in table format), with the authors stating that the intervention and treatment as usual (TAU) group differed on only one baseline characteristic (number of days in last 30 of activity limitation due to poor physical health, t = 1.96, df = 116, *p* = 0.05) which was controlled for in subsequent analyses. In addition, in Thompson et al. [35] the difference in PHQ-9 scores between intervention and TAU at baseline “approached significance (t = 1.88, df = 116, *p* = 0.062), with the mean for depressive symptoms in the intervention group (6.9), greater than that in the TAU group (5.5).”

Participants were recruited from a hospital-based epilepsy clinic [34], multiple clinical populations which the participating university sites had access to [35] and from a hospital’s neurology clinics [36]. Inclusion criteria were clearly presented by all studies. However, exclusion criteria were less well described by Thompson et al. [35]. Each of the studies incorporated quantitative measures only.

#### Design/aims of the studies

The three included studies were RCTs; both Thompson et al. [34] and Thompson et al. [35] employed a stratified randomised crossover design. The study by Thompson et al. [34] was a pilot, exploring the acceptability and efficacy of a Mindfulness-Based Cognitive Therapy (MBCT) based intervention (Project UPLIFT) in reducing depression, increasing knowledge, skills and self-efficacy and improving QOL among people with epilepsy and depression. Thompson et al. [35] aimed to evaluate the efficacy of the same intervention (Project UPLIFT with language modified for prevention) in reducing depressive symptoms and preventing major depressive disorder episodes (MDD) for people with epilepsy, and expanding its use to other geographical sites. Both Thompson et al. [34] and Thompson et al. [35] compared TAU with the delivery of the mindfulness course by either telephone or internet. The Tang et al. [36] assessor-blinded RCT aimed to investigate the effects of mindfulness-based therapy (MT) and social support (SS) for people with drug-resistant epilepsy, primarily the effect on QOL. The study involved MT plus SS as the “*active treatment condition*” and “*social support alone (SS) as an attention placebo control*”.

#### Mindfulness interventions

In the Thompson et al. [34] study the researchers “*created eight cognitive therapy and mindfulness sessions*”, targeted at people with epilepsy. The same intervention was utilised by Thompson et al. [35], with language modified to reflect preventing depressive symptoms in epilepsy. The interventions consisted of eight weekly sessions, lasting one hour [34, 35]. The MT in Tang et al. [36] involved four bi-weekly sessions lasting two and a half hours and was based on a protocol developed with reference to Kabat-Zinn [37], Hofmann et al. [38], Dahl et al. [39] and Kabat-Zinn [40]. None of the studies incorporated a day retreat which is a standard component in the most commonly used and researched MBI, Mindfulness-Based Stress Reduction (MBSR) (Table 3).

**Table 3:** Study description

Study	Country	Follow-up Period	Intervention
Thompson et al. (2010) [34]	USA	8 weeks	• Based on Mindfulness-Based Cognitive Therapy • Hour long sessions for eight weeks • Delivered via telephone or internet • Groups of six to eight participants • Facilitated by layperson with epilepsy and Master of Public Health Student Research Assistant – supervised by a licensed clinical psychologist. Mindfulness teaching certification/experience level not clear. • Sessions consisted of: check-in, instruction (video instruction – internet) skill building, discussion, homework assignment • Course content: attention to breath, sights and sounds; other meditations; body scan; progressive muscular relaxation; thought monitoring, identifying cognitive distortions, self-esteem, problem identification, goal-setting, identifying supports. • All participants had access to session materials and CD of relaxation and meditation exercises. Internet participants had access to online discussion forums. • Homework assignments given including monitoring thoughts, changing thoughts, practicing relaxation exercises, meditation exercises and mindfulness. Duration not specified.
Thompson et al. (2015) [35]	USA	10 weeks	• Based on Mindfulness-Based Cognitive Therapy, was script-based • Hour long sessions for eight weeks • Delivered via telephone or internet • 22 groups of up to seven participants • Facilitated by an adult with epilepsy and a graduate student with Mental Health Concentration in Public Health. Supervised by a licensed clinical psychologist and Associate Professor of Behavioural Sciences. Mindfulness teaching certification/experience level not clear, however four hours of training provided • Sessions consisted of: check-in period, teaching on topic of that week’s session, group discussion, skill-building exercise, homework assignment • Course content: knowledge about depression; monitoring and challenging, and changing thoughts; coping and relaxing; attention and mindfulness; focusing on pleasure; importance of reinforcement; preventing relapse. • Internet participants had access to archive of sessions and a discussion board. • Homework assignments given, duration not specified
Tang et al. (2015) [36]	China (Hong Kong)	6 weeks	• Mindfulness Therapy (experiential, progressive training on mindfulness techniques) plus social support • Four 2.5 hour bi-weekly sessions • Delivered in person • Groups of seven to eight participants • Facilitated by clinical psychologist. Mindfulness teaching certification/experience level not clear. • Course content: Knowledge and management of epilepsy; mind-body connection; Mindful breathing, eating, listening, observing; body scan; non-judgemental attitude, variation of thoughts, thought labelling. • All participants received an educational package on basic knowledge and management of epilepsy • 45 minutes of daily mindfulness practice encouraged. Homework assignments included recording thoughts and bodily sensation associated with recurrent seizure attack

#### Delivery

All three interventions were group-based, with group size ranging from six to eight. However, the MBCT interventions in Thompson et al. [35] and Thompson et al. [34] were delivered to participants in their own homes via telephone or the internet and were co-facilitated by a layperson with epilepsy and a Public Health Masters student research assistant or graduate student with a ‘Mental Health concentration in Public Health’, who were supervised by a licensed clinical psychologist. The Thompson et al. [35] intervention was manualised, and a script used to deliver the course online and by telephone – participants could interact in the telephone conferences or on the internet forum. In contrast, the MT in Tang et al. [36] was delivered face-to-face by a clinical psychologist.

Four hours of training was provided for facilitators in Thompson et al. [35] along with a practice session delivered by the Principal Investigator (a licensed clinical psychologist and Associate Professor of Behavioural Sciences, Psychiatry and Epidemiology). Training, qualifications and/or clinical experience of facilitators in delivering mindfulness therapy was not specified in Thompson et al. [34] and Tang et al. [36].

Participants in Thompson et al. [34] and Thompson et al. [35] were paid for their participation in the mindfulness course sessions ($15 per session) and the completion of study assessments ($25 per assessment). Participants in Tang et al. [36] did not receive any remuneration for taking part in the study.

#### Content

The mindfulness course piloted in Thompson et al. [34] provided information to increase knowledge about depression and epilepsy, incorporating both CBT and mindfulness techniques (Table 3). Mindfulness exercises included attention to breath, sights and sounds and other meditations; the intervention also utilised the body scan and progressive muscular relaxation (PMR). The same course was used as the basis of the intervention in Thompson et al. [35] “*with language modified for use in prevention*” of depressive episodes and reducing depressive symptoms. The study by Tang et al. [36] aimed to improve participants’ knowledge and management of epilepsy, with the course also providing training on mindfulness techniques - mindful breathing, −eating, −observing and the body scan. Mindfulness concepts such as the mind-body connection, awareness of thoughts, and a non-judgemental attitude, among others were also incorporated into the intervention.

#### Intervention materials

Intervention materials provided to participants were not clearly described across the studies. In Thompson et al. [34], participants receiving the intervention via the internet were provided with a manual on how to use the website. All participants were given access to session materials (although it is unclear what these constituted) and a CD containing relaxation and meditation exercises. Less information was provided in Thompson et al. [35], however session content was made available on an accessible archive for internet participants, who could also use an online discussion board. In Tang et al. [36], all participants were provided with an ‘educational package’ on epilepsy and its management.

#### Control group treatment

Participants in the attention placebo control group in Tang et al. [36] participated in a social support group with the same hours and group format as the mindfulness intervention. The first session of the group focused on psycho-education concerning epilepsy, and the remaining three sessions provided a supportive environment for sharing and reinforcing epilepsy management. Control participants in Thompson et al. [34] and Thompson et al. [35] received TAU and continued with their usual medication and/or therapy, but this was not clearly described. Due to the crossover design of these studies, control participants also received the mindfulness intervention.

### Outcomes

#### Data collection

Data were collected at three time points in Thompson et al. [34] and Thompson et al. [35] – pre-test, interim (after the end of the MBCT eight week intervention) and post-assessment (after the end of the TAU intervention). Two data collection points were utilised in Tang et al. [36], baseline (including six-week prospective baseline period) and post-intervention – (six weeks following the last intervention session). Only Tang et al. [36] described performing a sample size calculation (60 participants), statistical power (0.8) and effect size (0.74) to detect change in QOL scores (QOLIE-31-P). Thompson et al. [35] noted an effect size (*r* = 0.20) to detect change in depression symptoms, while Thompson et al. [34] stated that they “*designed and powered this study to detect change in depressive symptoms over 8 weeks using the BDI*” (Beck Depression Inventory) without further detail (Table 4).

**Table 4:** Study outcomes and main findings

Author (Year)	Main outcomes	Main findings
Thompson et al. (2010) [34]	Depressive Symptoms (Beck Depression Inventory and Modified form) Self-Efficacy (Depression Self-Efficacy Scale DCSES) Satisfaction with life (Satisfaction With Life Scale SWLS) Quality of Life (Behavioural Risk Factor Surveillance System BRFSS) Self-Compassion (Self-Compassion Scale SCS) Knowledge and Skills assessment	Decrease in depressive symptoms among intervention group significantly better than control, and greater for those attending more sessions. No statistical difference in efficacy in relation to presence of MDD. Telephone group results slightly better than internet group but not significant. Intervention group increased knowledge and skills more than control group; and increases were greater among those who attended more sessions. Change in knowledge and skills were negatively correlated with change in BDI score. Change in Satisfaction with Life approached significance. No significant improvement in physical and mental health QOL measures, but improvement greater in intervention group.
Thompson et al. (2015) [35]	Depressive Symptoms (Beck Depression Inventory and Modified form; Neurological Disorders Depression Inventory for Epilepsy NDDI-E; PHQ-9) Depression Coping Self-Efficacy (Depression Coping Self-Efficacy Scale DCSES) Self-Compassion (Self-Compassion Scale SCS) Satisfaction with Life (Satisfaction With Life Scale SWLS) Quality of Life (Behavioural Risk Factor Surveillance System) Knowledge and Skills assessment	Depressive symptoms – no difference between telephone and internet groups. Significant improvement in intervention group and had less depressive episodes. Association between scores and number of sessions attended. Knowledge and skills improvements greater in intervention group, and associated with number of sessions attended. Changes significantly associated with change in mBDI scores. Knowledge and skills mediated change in mBDI scores between intervention and controls. Satisfaction with life increased in intervention group, and associated with number of sessions attended. Changes in Depression Coping Self Efficacy and Physical and Mental Health Quality of Life and Self-Compassion not significant.
Tang et al. (2015) [36]	Quality of Life (Patient-Weighted Quality of Life in Epilepsy Inventory QOLIE-31-P) Depressive Symptoms (Beck Depression Inventory-II, BDI-II) Anxiety Symptoms (Beck Anxiety Inventory, BAI)	Control and intervention group statistically significant improvement in quality of life and anxiety scores. Improvements statistically significantly better in intervention group. Statistically significant reduction in BDI-II scores in both groups, not clinically significant. Significant reduction in seizure frequency and severity in both groups. Significant improvement in verbal and non-verbal memory. No other differences in Cognitive Functioning.

*BDI* Beck Depression Inventory, *MDD* Major Depressive Disorder, *mBDI* modified Beck Depression Inventory, *QOL* Quality of Life

#### Epilepsy measures

In Tang et al. [36] participants were required to keep a seizure diary during the span of the study and to complete a Seizure Severity Questionnaire. Significant reductions were found in frequency of seizures (F = 25.51, *p* < 0.001 hp2 = 0.306, 95% CI −3.96, −1.64) with the group-by-time interaction being statistically significant (F = 5.90, *p* = 0.018, hp2 = 0.092). In addition, there was a statistically significant reduction in severity of seizures (F = 15.28, *p* < 0.001, hp2 = 0.209, 95% CI −0.91, −0.29), although there was no significant difference between the two groups. Studies Thompson et al. [34] and Thompson et al. [35] did not measure epilepsy-related outcomes such as seizure frequency.

#### Depression

All of the studies utilised variations of the Beck Depression Inventory (BDI): Beck Depression Inventory-II (BDI-II) [36]; BDI and modified BDI (mBDI) [34, 35]. In addition to these measures, Thompson et al. [34] included the Depression Coping Self-Efficacy Scale (DCSES), whilst Thompson et al. [35] incorporated the DCSES, the Neurological Disorders Depression Inventory for Epilepsy (NDDI-E) and PHQ-9 (which was initially used to exclude participants with baseline scores indicative of MDD).

Both Thompson et al. [34] and Thompson et al. [35] reported significant reductions in depressive symptoms among intervention participants. Thompson et al. [34] found statistically significant reductions in BDI (F_1,37_ = 11.99, *p* = 0.001) and mBDI (F_1,37_ = 10.08, *p* = 0.003) scores. Similarly Thompson et al. [35] found statistically significant reductions in BDI (F_1,106_ = 4.50, p = 0.036) and mBDI (F_1,106_ = 4.67, p = 0.033); the study also found that intervention participants experienced fewer depressive episodes than TAU participants (p = 0.028). Both studies reported a dose-response relationship, with participants who attended more sessions, experiencing the largest reductions (F_2,35_ = 1.53, *p* = 0.23 [not statistically significant] [34]). In terms of the outcomes for participants in either the telephone or internet groups, Thompson et al. [35] found no difference based on allocation (mBDI: F_1,50_ = 0.02, *p* = 0.88), whilst Thompson et al. [34] found that depressive symptoms decreased more among the telephone group, however this difference was not significant.

The intervention group in Thompson et al. [35] were also found to have a greater decrease in NDDI-E scores, but this was not significant (F_1,106_ = 0.35, *p* = 0.555). No significant difference was found between groups on the DCSES in Thompson et al. [34] (F_1,37_ = 2.14, *p* = 0.152) and Thompson et al. [35]. In addition, Thompson et al. [34] suggested that the intervention was “*equally effective regardless of anti-depressant medication or psychotherapy”* (however study was not powered to detect this).

Thompson et al. [35] reported that participants in the intervention group were less likely to experience an incidence of MDD than those in the TAU group (*p* = 0.028). In addition, a greater decrease in depressive symptoms was found among the intervention group than the TAU group which approached significance for the PHQ-9 (F_1,106_ = 2.75, *p* = 0.100), and when the analysis was limited to those who provided both baseline and interim data the results were significant (F_1,104_ = 3.895, *p* = 0.050).

In Tang et al. [36] there was a statistically significant reduction in depressive symptoms measured by BDI-II scores in both groups after treatment. However, there was no statistically significant difference on severity category pre- and post-intervention (*p* = 0.125 both groups). This implies that the improvement in BDI-II scores was of no clinical significance.

#### Anxiety

Only Tang et al. [36] measured anxiety, using the Beck Anxiety Inventory (BAI). Both the MT and SS groups showed a statistically significant reduction in anxiety scores post-intervention (F = 23.44, *p* < 0.001, hp2 = 0.288, 95% CI −6.44, −1.76). Using McNemar tests, it was shown that there was a clinically significant reduction in the MT group (*p* = 0.012) but not the SS group (*p* = 0.065) between pre-and post-intervention.

#### Quality of Life measures

An epilepsy specific QOL measure was used in Tang et al. [36] - Quality of Life in Epilepsy Inventory (QOLIE-31-P). The Satisfaction with Life Scale (SLS) and Behavioural Risk Factor Surveillance System (BRFSS) were used in Thompson et al. [34] and Thompson et al. [35]. Satisfaction with life improved significantly in the intervention group compared with the control group in Thompson et al. [35] (F_1,106_ = 8.02, *p* = 0.006), associated with number of sessions attended, while the improvement in Thompson et al. [34] approached significance (F_1,37_ = 3.029, *p* = 0.090). In both studies physical and mental health QOL measures improved (most for the intervention group in Thompson et al. [34]), but were not statistically significant. In contrast, Tang et al. [36] found statistically significant improvement in QOL in both the intervention and SS control group (F_1,58_ = 30.35, *p* < 0.001, hp2 = 0.334, 95% CI +3.38, +7.24), with the improvement in the intervention group being clinically important in more participants and statistically significantly better (*χ* ^2^ (1) = 4.356, *p* = 0.037, φ = 0.269). Other aspects of QOL were also found to have improved including energy, mood, medication effect and seizure worry.

Thompson et al. [35] also measured self-compassion using the Self-Compassion Scale (SCS) but found no significant change.

#### Knowledge and Skills

Change in knowledge and skills relating to depression were assessed in Thompson et al. [35] and Thompson et al. [34] using the same measure, originally developed for use in the earlier study. Both reported a statistically significant improvement in scores (F_1,37_ = 4.75, *p* = 0.036 [34]; F_1,106_ = 6.01, *p* = 0.016 [35]) with improvements greater for those attending more sessions, however Thompson et al. [34] noted that this was not statistically significant (F_2,35_ = 0.47, *p* = 0.63).

According to Thompson et al. [34], a change in knowledge and skills score was “*significantly negatively correlated with change in BDI score (r = −0.389, p = 0.01*3)”. In addition, mBDI scores were found to be significantly associated with changes in knowledge and skills scores (*r* = −0.30, *p* = 0.002) in Thompson et al. [35]. This study also found that change in knowledge and skills mediated the relationship between change in mBDI scores in the control and intervention groups, which was evaluated using structural equation modelling.

#### Cognitive functioning

Cognitive functioning was assessed using a number of measures in Tang et al. [36]. These included: Chinese Auditory Verbal Learning Test; Rey Complex Figure Test and Recognition Trial; Category Fluency Test; Digit Span Test; Stroop Colour and Word Test – Victoria version. Improvements were found in verbal and non-verbal memory; no differences were reported for fluency, Digit Span Test and Stroop Colour and Word Test. However, the authors noted that practice effects could have affected these findings and so should be treated with caution. Cognitive functioning was not assessed by Thompson et al. [34] and Thompson et al. [35].

### Quality appraisal

Using the Cochrane Collaboration ‘Risk of Bias’ tool (Table 5), studies by Thompson et al. [34] and Thompson et al. [35] were assessed as being at unclear/high risk of bias; the study by Tang et al. [36] was assessed as being at low risk. Thompson et al. [34] was considered to be at high risk of performance and detection bias – “*neither the participants nor the project staff were blinded to the group assignment*”. The study was also judged as being at high risk of attrition bias because of missing data not being described and further due to the study’s repeated measures design, only participants who completed interim assessments were included in analyses. The Thompson et al. [35] study was at high risk of selection bias as the randomisation and allocation procedures were not described and some participants randomised to the intervention group were able to choose whether to receive the intervention by phone or internet.

**Table 5:** Cochrane risk of bias table

	Thompson et al. (2010) [34]		Thompson et al. (2015) [35]		Tang et al. (2015) [36]	
Entry	Judgement	Support for judgement	Judgement	Support for judgement	Judgement	Support for judgement
Random sequence generation (selection bias)	Unclear Risk	Quote: “Using a stratified randomized, crossover design we randomly assigned participants to one of four strata.” Randomisation method is not described	Unclear Risk	Quote: “Using a stratified randomized, crossover design we randomly assigned participants to one of four strata.” Randomisation procedure not described	Low Risk	Quote: “Simple randomization by drawing was performed within each block to assign patients to one of the groups alternatively” Random allocation
Allocation concealment (selection bias)	Unclear Risk	Unclear whether participants/assessors could have been aware of allocation to intervention/control in advance	High Risk	Quote: “…within each condition, people who required a particular mode of delivery (web or telephone) were placed in that group and the remainder (the majority of participants) were assigned to equalise the groups.” Not described, appears patients were allocated to different interventions (web or telephone) based on preference/need	Unclear Risk	Quote: “randomization was performed by an independent research assistant” Randomization performed by independent research assistant but method of concealment not explained.
Blinding of participants and personnel (performance bias)	High Risk	Quote: “Neither the participants nor the project staff were blinded to the group assignment.” Clearly stated in paper under 2.5.3 Recruitment that neither were blinded	High Risk	Not described, and as above participants could choose to receive telephone or web delivery.	Low Risk	Quote: “A team of trained research assistants…who were blinded to participants’ intervention group performed all assessments…” The assessors were blinded to the patient’s intervention grouping.
Blinding of outcome assessment (detection bias)	High Risk	Quote: “Neither the participants nor the project staff were blinded to the group assignment.” Clearly stated in paper under 2.5.3 Recruitment that neither were blinded	Unclear Risk	Not described	Low Risk	Quote: “…team of trained research assistants with a bachelor’s degree in psychology who were blinded to participants’ intervention group performed all assessments; they were separated into 2 teams, one for baseline assessment and the other for post intervention assessment.”
Incomplete outcome data addressed (attrition bias)	High Risk	Due to repeated measures design only participants completing interim assessments were included in analyses. 13 participants left the study, not clear if control or intervention. Missing data not described	Unclear Risk	Attrition poorly characterised. Attendance poorly described including reasons for not attending. Missing values were imputed, but levels of missing values were not described. However intention-to-treat analysis carried out according to participants’ original treatment assignment.	Low Risk	Missing outcome data balanced in numbers across intervention groups.
Selective reporting (reporting bias	Unclear Risk	All outcomes appear to have been reported, however no protocol.	Unclear Risk	Primary outcome not defined and poor discussion of four of six main outcomes (DCSE, SC, Physical and mental health QoL). No protocol.	Low Risk	Full study protocol is available
Other Bias	Unclear Risk		Unclear Risk		Low Risk	No other sources of bias

## Discussion

This review identified three studies which reported the findings of RCTs utilising MBIs in the treatment of people with epilepsy. The primary outcomes assessed in the three included papers were depression [34, 35] and QOL [36] and each reported that there may be some benefit associated with the use of MBIs for people with epilepsy. Thompson et al. [34] and Thompson et al. [35] found significant reductions in depressive symptoms. In relation to QOL, Tang et al. [36] reported a statistically significant improvement; Thompson et al. [35] also reported an improvement in QOL. Other outcomes also found to have improved following an MBI included anxiety [36] and depression knowledge and skills [34, 35].

This review included only three articles for final data extraction which limits the applicability of the findings. Despite positive findings, their generalisability and reliability are limited by several factors. As previously noted two of the studies were at unclear/high risk of bias - randomisation and allocation procedures, adverse events and reasons for drop-outs were poorly reported. Furthermore, two of the papers were described as being underpowered - Thompson et al. [34] highlighted that their study was not sufficiently powered to find statistically significant differences in satisfaction with life scores. Moreover, Thompson et al. [35] noted that, as the study was powered to identify any differences in depressive symptoms, it was limited in power to detect *“smaller differences seen in self-efficacy and self-compassion, or changes seen at follow-up*”. In addition, all studies noted their results and generalisability were limited by taking place in just one site [34, 36], or over a small number of sites [35]. All studies also highlighted their short follow-up periods and lack of measurement of longer-term effects. Tang et al. [36] also noted possible limited accounting for confounding factors such as anti-epileptic medications and other uncontrolled variables. In addition participants’ home practice was not measured across the studies.

Demographic and epilepsy characteristics of participants were poorly reported in Thompson et al. [34] and Thompson et al. [35] limiting the extent to which the role of these factors could be examined. Furthermore, all participants in Tang et al. [36] had drug-resistant epilepsy; it is therefore unclear whether the same outcomes could be expected among those whose epilepsy is not drug-resistant. In addition, as the included studies utilised different variations of MBIs, caution should be used in comparing results across studies.

To our knowledge no other systematic reviews have focused on the use of MBIs alone for people with epilepsy. However other systematic reviews have included a wider range of psychological treatments, some involving aspects of mindfulness. A review of psychological treatments for epilepsy (including relaxation therapies, CBT and educational interventions) suggested there may be “*a possible beneficial effect on seizure frequency*” while “*no reliable conclusions*” could be reached on the effects of psychological treatments on psychological outcomes and QOL [41] (has been recalled to be superseded). A further review [22] examined treatments (antidepressant medications, antiepileptic medications and CBT - including Thompson et al. [34]) for people with comorbid epilepsy and depression, and suggested CBT could improve health outcomes although a number of methodological limitations were identified and questions raised about implementation.

As the scope of this review was restricted to MBIs, interventions that included only some aspects of mindfulness were excluded. For example, Acceptance and Commitment Therapy (ACT) and Yoga for patients with drug-refractory epilepsy have been found to improve seizure frequency and QOL in RCTs [42, 43]. Yoga interventions were also excluded from the review; however a study by Panjwani et al. [44] suggested that Sahaja Yoga practice reduced stress levels among participants with epilepsy.

The findings of this review were limited due to the small number and poor quality of studies included. It was not possible to conduct a meta-analysis and results were therefore presented in a narrative format. In addition, we were unable to locate the full paper for one study at the full paper screening stage – the paper was therefore excluded. Furthermore, for pragmatic reasons our search strategy did not include an extensive grey literature search. However we did conduct a basic (as opposed to advanced) search of Google and searched electronic databases that index grey literature items. A strength is that as previously noted, we are not aware of another review focusing on the use of MBIs for people with epilepsy and it has therefore been valuable in determining the extent of existing research in this area.

## Conclusions

Further research is required before conclusions can be reached on the effectiveness of mindfulness as a therapeutic intervention for people with epilepsy. In order to establish longer-term outcomes of participation in MBIs, larger RCTs with longer follow-up periods and active control groups are required along with more detailed information relating to participants’ epilepsy.

## Additional file

Additional file 1: MEDLINE with Full Text (OVID) search strategy. (DOCX 13 kb)

## Abbreviations

ACT: Acceptance and commitment therapy
BAI: Beck anxiety inventory
BDI: Beck depression inventory
BDI-II: Beck depression inventory II
BRFSS: Behavioural risk factor surveillance system
CBT: Cognitive behavioural therapy
DCSES: Depression coping self efficacy scale
LTC: Long term condition
MBCT: Mindfulness based cognitive therapy
mBDI: Modified beck depression inventory
MBI: Mindfulness based intervention
MBSR: Mindfulness Based Stress Reduction
MDD: Major depressive disorder
MT: Mindfulness therapy
NDDI-E: Neurological disorders depression inventory for epilepsy
PICO: Population, intervention, comparator, outcomes
PMR: Progressive muscular relaxation
QOL: Quality of life
QOLIE-31-P: Quality of life in epilepsy inventory
RCT: Randomised controlled trial
SCS: Self-compassion scale
SLS: Satisfaction with life score
SS: Social support
TAU: Treatment as usual

## References

[1] Jones JE, Hermann BP, Barry JJ, Gilliam FG, Kanner AM, Meador KJ (2003). Rates and risk factors for suicide, suicidal ideation, and suicide attempts in chronic epilepsy. Epilepsy Behav.

[2] Kanner AM (2003). Depression in epilepsy: prevalence, clinical semiology, pathogenic mechanisms, and treatment. Biol Psychiatry.

[3] Beyenburg S, Mitchell AJ, Schmidt D, Elger CE, Reuber M (2005). Anxiety in patients with epilepsy: systematic review and suggestions for clinical management. Epilepsy Behav.

[4] Kanner AM (2006). Depression and epilepsy: a new perspective on two closely related disorders. Epilepsy Curr.

[5] Jackson MJ, Turkington D (2005). Depression and anxiety in epilepsy. J Neurol Neurosurg Psychiatry.

[6] Fiest KM, Dykeman J, Patten SB, Wiebe S, Kaplan GG, Maxwell CJ, Bulloch AG, Jette N (2013). Depression in epilepsy: a systematic review and meta-analysis. Neurology.

[7] Lee SA, Lee SM, No YJ (2010). Factors contributing to depression in patients with epilepsy. Epilepsia.

[8] Nakken KO, Solaas MH, Kjeldsen MJ, Friis ML, Pellock JM, Corey LA (2005). Which seizure-precipitating factors do patients with epilepsy most frequently report?. Epilepsy Behav.

[9] Temkin NR, Davis GR (1984). Stress as a risk factor for seizures among adults with epilepsy. Epilepsia.

[10] Betts T (1992). Epilepsy and stress. BMJ.

[11] Haut SR, Vouyiouklis M, Shinnar S (2003). Stress and epilepsy: a patient perception survey. Epilepsy Behav.

[12] Maguire J, Salpekar JA (2013). Stress, seizures, and hypothalamic-pituitary-adrenal axis targets for the treatment of epilepsy. Epilepsy Behav.

[13] de Souza EA, Salgado PC (2006). A psychosocial view of anxiety and depression in epilepsy. Epilepsy Behav.

[14] Yuen AW, Thompson PJ, Flugel D, Bell GS, Sander JW (2007). Mortality and morbidity rates are increased in people with epilepsy: is stress part of the equation?. Epilepsy Behav.

[15] Kerr MP (2012). The impact of epilepsy on patients’ lives. Acta Neurol Scand Suppl.

[16] Fisher RS, Vickrey BG, Gibson P, Hermann B, Penovich P, Scherer A, Walker S (2000). The impact of epilepsy from the patient’s perspective I. Descriptions and subjective perceptions. Epilepsy Res.

[17] Harden CL (2002). The co-morbidity of depression and epilepsy: epidemiology, etiology, and treatment. Neurology.

[18] Hermann BP, Seidenberg M, Bell B (2000). Psychiatric comorbidity in chronic epilepsy: identification, consequences, and treatment of major depression. Epilepsia.

[19] Kimiskidis VK, Valeta T (2012). Epilepsy and anxiety: epidemiology, classification, aetiology, and treatment. Epileptic Disord.

[20] Gilliam F, Kanner AM (2002). Treatment of depressive disorders in epilepsy patients. Epilepsy Behav.

[21] Tang V, Michaelis R, Kwan P (2014). Psychobehavioral therapy for epilepsy. Epilepsy Behav.

[22] Mehndiratta P, Sajatovic M (2013). Treatments for patients with comorbid epilepsy and depression: a systematic literature review. Epilepsy Behav.

[23] Gandy M, Sharpe L, Perry KN (2013). Cognitive behavior therapy for depression in people with epilepsy: a systematic review. Epilepsia.

[24] Simpson R, Booth J, Lawrence M, Byrne S, Mair F, Mercer S (2014). Mindfulness based interventions in multiple sclerosis - a systematic review. BMC Neurol.

[25] Lawrence M, Booth J, Mercer S, Crawford E (2013). A systematic review of the benefits of mindfulness-based interventions following transient ischemic attack and stroke. Int J Stroke.

[26] Kabat-Zinn J (1990). Full catastrophe living: the program of the stress reduction clinic at the university of Massachusetts medical centre.

[27] Segal ZV, Williams JMG, Teasdale JD. Mindfulness- Based Cognitive Therapy for Depression. Second edn. New York: Guilford Press; 2012.

[28] Piet J, Hougaard E (2011). The effect of mindfulness-based cognitive therapy for prevention of relapse in recurrent major depressive disorder: a systematic review and meta-analysis. Clin Psychol Rev.

[29] Hofmann SG, Sawyer AT, Witt AA, Oh D (2010). The effect of mindfulness-based therapy on anxiety and depression: a meta-analytic review. J Consult Clin Psychol.

[30] Fjorback LO, Arendt M, Ørnbøl E, Fink P, Walach H (2011). Mindfulness-based stress reduction and mindfulness-based cognitive therapy – a systematic review of randomized controlled trials. Acta Psychiat Scand.

[31] Lustyk M, Chawla N, Nolan R, Marlatt G (2009). Mindfulness meditation research: issues of participant screening, safety procedures, and researcher training. Adv Mind Body Med.

[32] Wood K, Lawrence M, Jani B, Simpson R, Mercer SW: Mindfulness based interventions in epilepsy. 2016. http://www.gla.ac.uk/researchinstitutes/healthwellbeing/research/generalpractice/research/mbsr-epilepsy/. Accessed 9 Dec 2016 10.1186/s12883-017-0832-3 PMC5360054 28320349

[33] Higgins JPT, Green S, editors. Cochrane Handbook for Systematic Reviews of Interventions Version 5.1.0. 2011. [updated March 2011] http://www.handbook.cochrane.org. Accessed 15 Mar 2016.

[34] Thompson NJ, Walker ER, Obolensky N, Winning A, Barmon C, DiIorio C, Compton MT (2010). Distance delivery of mindfulness-based cognitive therapy for depression: project UPLIFT. Epilepsy Behav.

[35] Thompson NJ, Patel AH, Selwa LM, Stoll SC, Begley CE, Johnson EK, Fraser RT (2015). Expanding the efficacy of project UPLIFT: distance delivery of mindfulness-based depression prevention to people with epilepsy. J Consult Clin Psychol.

[36] Tang V, Poon WS, Kwan P (2015). Mindfulness-based therapy for drug-resistant epilepsy: an assessor-blinded randomized trial. Neurology.

[37] Kabat-Zinn J (2003). Mindfulness-based interventions in context: past, present, and future. Clin Psychol.

[38] Hofmann SG, Sawyer AT, Fang A (2010). The empirical status of the “New wave” of CBT. Psychiatr Clin North Am.

[39] Dahl JLT, McCracken LM (2011). Analysis and treatment of epilepsy using mindfulness, acceptance, values, and countermeasures. Mindfulness and acceptance in behavioral medicine.

[40] Kabat-Zinn J (2006). Coming to our senses: healing ourselves and the world through mindfulness.

[41] Ramaratnam S, Baker GA, Goldstein LH. Psychological treatments for epilepsy. Cochrane Database Syst Rev. 2008(3):Cd002029. 10.1002/14651858.CD002029.pub3 10.1002/14651858.CD002029.pub3 18646083

[42] Lundgren T, Dahl J, Melin L, Kies B (2006). Evaluation of acceptance and commitment therapy for drug refractory epilepsy: a randomized controlled trial in South Africa--a pilot study. Epilepsia.

[43] Lundgren T, Dahl J, Yardi N, Melin L (2008). Acceptance and commitment therapy and yoga for drug-refractory epilepsy: a randomized controlled trial. Epilepsy Behav.

[44] Panjwani U, Gupta H, Singh S, Selvamurthy W, Rai U (1995). Effect of Sahaja yoga practice on stress management in patients of epilepsy. Indian J Physiol Pharmacol.

[45] Carmody J, Baer RA (2009). How long does a mindfulness-based stress reduction program need to be? A review of class contact hours and effect sizes for psychological distress. J Clin Psychol.

