# Supplementary material for: Mindfulness-based interventions in epilepsy: a systematic review
Source: BMC Neurol. 2017 Mar 20;17:52. doi: 10.1186/s12883-017-0832-3 (PMC5360054; doi:10.1186/s12883-017-0832-3)
Supplement: Additional file 1: — MEDLINE with Full Text (OVID) search strategy. (DOCX 13 kb) [file 12883_2017_832_MOESM1_ESM.docx]

**Appendix 1**

**MEDLINE with Full Text** (**OVID)** **search strategy**

1. exp Epilepsy/

2. exp Seizures/

3. epilep$

4. seizure$

5. convuls$

6. 1 or 2 or 3 or 4 or 5

7. exp Meditation/

8. exp Relaxation Therapy/

9. exp Relaxation/

10. exp Breathing Exercises/

11. exp Mindfulness/

12. Mindfulness based stress reduction.mp.

13. Mindfulness based cognitive therapy.mp.

14. Mindfulness based intervention*

15. MBSR or MBCT

16. Meditation

17. Relaxation therapy

18. (Relaxation adj2 (technique* or therap*))

19. (Breathing adj2 (technique* or therap*))

20. 7 or 8 or 9 or 10 or 11 or 12 or 13 or 14 or 15 or 16 or 17 or 18 or 19

S16. S5 and S15

S17. S5 and S15 Limiters - Date of Publication from: 19800101-20161231; Age Related: All Adult: 19+ years; Languages: English; Human
